# The Relationship between Feelings-of-Knowing and Partial Knowledge for General Knowledge Questions

**DOI:** 10.3389/fpsyg.2016.00996

**Published:** 2016-06-29

**Authors:** Elisabeth Norman, Oskar Blakstad, Øivind Johnsen, Stig K. Martinsen, Mark C. Price

**Affiliations:** ^1^Department of Psychosocial Science, Faculty of Psychology, University of Bergen, BergenNorway; ^2^Explorable AS, KristiansandNorway

**Keywords:** metacognition, feeling-of-knowing, recall, accessibility hypothesis, partial knowledge, working memory

## Abstract

*Feelings of knowing (FoK)* are introspective self-report ratings of the felt likelihood that one will be able to recognize a currently unrecallable memory target. Previous studies have shown that FoKs are influenced by retrieved fragment knowledge related to the target, which is compatible with the *accessibility hypothesis* that FoK is partly based on currently activated partial knowledge about the memory target. However, previous results have been inconsistent as to whether or not FoKs are influenced by the accuracy of such information. In our study (*N* = 26), we used a *recall-judge-recognize* procedure where stimuli were general knowledge questions. The measure of partial knowledge was wider than those applied previously, and FoK was measured before rather than after partial knowledge. The accuracy of reported partial knowledge was positively related to subsequent recognition accuracy, and FoK only predicted recognition on trials where there was correct partial knowledge. Importantly, FoK was positively related to the amount of correct partial knowledge, but did not show a similar incremental relation with incorrect knowledge.

## Introduction

In everyday cognition, metamemory judgments may occur when we fail to retrieve information from memory, but where the accompanying subjective feeling indicates *potential* access: it feels that with more time, or under different circumstances, one might have been able to retrieve the searched-for information after all. A common example of this form of experience is the feelings of knowing (FoK, Brown, 1991; Koriat, 1993, 1995, 2007). Feelings of this type can be studied using the recall-judge-recognize (RJR) procedure (Hart, 1965). For items that are not successfully retrieved during an initial recall phase, participants rate the felt likelihood of being able to recognize the correct item among a set of alternatives. FoKs are then compared with subsequent recognition performance. This procedure can be used with factual questions relating to existing general knowledge, or with knowledge that is newly acquired in the laboratory. Across a variety of studies using different experimental procedures, FoKs have proven a good predictor of recognition memory performance (Metcalfe, 2000), although there is disagreement over their exact nature. In line with the focus of this research topic, this paper aims to get a better insight into to what extent FoKs reflect the amount of relevant information currently activated in working memory.

It has been variously suggested that FoK (a) reflects a direct monitoring of memory traces (the *trace access view*; see Hart, 1965), (b) involves fast monitoring of the familiarity of the stimuli used to prompt memory retrieval, e.g., familiarity of knowledge domains referred to by words in a general knowledge question (the *cue familiarity hypothesis*; see Reder and Ritter, 1992; Metcalfe et al., 1993), or (c) reflects products of the ongoing retrieval process such as the quantity and salience of partial or fragmentary target information retrieved during memory search (the *accessibility hypothesis*; see Koriat, 1993, 1995). Some broader models acknowledge separate influences of different mechanisms. According to one such model (Koriat and Levy-Sadot, 2001) FoKs are initially shaped by cue familiarity but are subsequently also influenced by the relative accessibility of partial knowledge that comes to mind during the retrieval attempt. Another integrative hypothesis (Liu et al., 2007) is that positive FoK judgments (“knowing that you know”) are based on partial recovery of non-recalled targets, and negative FoK judgments (“knowing that you do not know”) on cue familiarity.

One way of studying the accessibility hypothesis is to ask participants within a traditional RJR experiment to report any fragmentary knowledge of the target even when they are unable to correctly retrieve the target itself, which has been referred to as *overt accessibility* of partial knowledge (Koriat, 1995). The classic series of studies to explore this is a series of experiments by Koriat (1993, 1995). Stimuli were either general knowledge questions, nonsense letter strings, or nonsense word pairs. In all experiments, FoK was positively related to the total amount of partial knowledge reported, regardless of its accuracy. This is consistent with the proposal that FoK is causally based on the cumulative amount of partial knowledge that people feel they have accessed. However, more recent studies have shown that FoKs may be more strongly influenced by correct than incorrect partial information (Dunlosky et al., 2005; Thomas et al., 2011, 2012). However, both Dunlosky et al. (2005) and Thomas et al. (2011, 2012) studied newly acquired rather than pre-existing knowledge. More specifically, stimuli included recently acquired factual knowledge (Dunlosky et al., 2005), as well as pairs of words, pictures, or meaningless figures (Thomas et al., 2011, 2012). Neither of the studies looked at the relationship between fragment knowledge and FoK for pre-existing semantic knowledge. The generalizability of these findings to memory situations involving general knowledge questions, is therefore unclear.

Moreover, in studies involving general knowledge questions (Koriat, 1993, 1995), measures of partial knowledge were quite narrowly constrained in that people were specifically asked to report structural fragments of the target (e.g., any letters recalled). This might underestimate the contribution to FoK from other types of partial knowledge. In fact, Koriat (1995, p. 312) acknowledged the possible influences of “semantic attributes, episodic information pertaining to the target, and activations emanating from other sources.” Accurate measurement of these other types of partial knowledge would be especially important when stimuli were general knowledge questions measuring pre-existing knowledge, rather than newly acquired knowledge of meaningless stimuli (e.g., non-word letter strings). Furthermore, a narrowly constrained measure of partial recall has a second disadvantage. When the partial recall measurement involves selecting items from a predefined set of alternatives, as in Koriat’s (1993, 1995) studies, it is easier for participants to spuriously generate guessed partial knowledge than with an open response format. It should also be noted that in Koriat’s (1993, 1995) studies, as well as in all but one of the studies reported by Thomas et al. (2011, 2012), FoK ratings were given *after* partial knowledge had been reported on any given trial. With this procedure, one cannot rule out the possibility that FoK ratings were based on a simple count of the amount of reported partial knowledge, regardless of whether this knowledge was correct or incorrect, and regardless of whether or not the incorrect partial knowledge was genuinely felt to be correct or was prompted by the ease of generating spurious examples.

In the current study, we address whether FoKs reflect partial knowledge in a situation where FoKs and memory were measured for general knowledge questions, and where FoK was measured before the retrieval attempt. Our RJR procedure differed from Koriat’s (1993, 1995) procedure in two important ways. First, our procedure for measuring partial knowledge was more open-ended in that the only constraint given to participants was a limit on the maximum number of items they could report. A positive relationship between FoK and partial knowledge would support the hypothesis that FoK reflects overt accessibility. Conversely, the lack of such relationship would suggest that FoK might be more affected by other types of cues. Because our procedure was designed to reflect a broader array of partial knowledge on which naturally occurring FoKs are likely to be based, we also predicted that recognition accuracy would now be positively related to the accuracy of partial knowledge, as in the studies by Dunlosky et al. (2005) and Thomas et al. (2011, 2012). To reduce the likelihood that FoK ratings were just based on explicit evaluations of the amount of partial knowledge that was deliberately retrieved and reported, and therefore improve the ecological validity of the FoK paradigm, our experiment also measured FoK immediately after recall failure but *before* reports of partial knowledge. In addition, following Schwartz et al. (2015) we measured FoK on all trials, not just following retrieval failure.

## Materials and Methods

### Participants

Twenty-six student volunteers (16 women and 10 men) aged 19–27 years (*M* = 20.8) were rewarded to take part for approximately 1.5 h with a lottery for gift tokens. The research was conducted in accordance with the stipulations in the declaration of Helsinki, and conformed to the regulations of the Norwegian Data Protection Official for Research. Since participation was anonymous and the data was therefore not covered by the Norwegian Personal Data Act, the study was exempt from the requirement to formally apply to the Data Protection Official for Research. Since the study was not related to health, it was also not covered by the Norwegian Health Research Act and was exempt from the requirement to formally apply to the Regional Committee for Medical and Health Research Ethics. Participants signed a written informed consent form in advance of participation, in accordance with the official guidelines from the Declaration of Helsinki.

### Design

On each trial of a recall phase we consecutively tested (a) free recall of the answer to a general knowledge question, (b) self-rated FoK that the correct answer could be recognized later in a multiple-choice test, (c) report of any partial knowledge, and (d) a second chance to report the answer in case conservative response biases inhibited overt recall during the first free recall opportunity. On each trial of a subsequent recognition phase, the correct answer to a previous question had to be selected from among distracters.

The main independent variable (within-subjects) was the quantity and accuracy of reported partial knowledge, and the main dependent variables were FoK ratings, FoK accuracy, and recognition accuracy.

### Apparatus and Stimulus Material

Participants were tested in groups. Stimulus materials were Norwegian. Pre-recorded instructions were played over speakers. General knowledge questions for the initial recall task were presented both via speakers and visually on a lecture hall screen using E-prime (Schneider et al., 2002a,b). Participants responded in a booklet where each question had a separate area for recall, FoK, partial knowledge and second recall responses. For the recognition phase, multiple-choice questions were presented in a separate booklet where participants selected responses.

### Procedure

Each of 64 recall trials started with a general information question (e.g., “What is the name of the film that obtained seven Academy Award nominations in 1999?”) with 10 s for written responding. If no answer was given, participants were to draw a line through the response box to prevent completing the answer later. An auditory prompt then invited a FoK rating on a 10-point scale [from weak (1) to strong (10)], and within 8 s, to indicate the perceived likelihood of recognizing the correct answer if this was presented among several alternatives. Partial knowledge was then reported. Instructions were to write down any related knowledge within 20 s, but not more than six units of knowledge (indicated by six numbered lines in the response booklet). One example question (“What is the name of the American who won Tour de France 6 times?”) and three suggested partial knowledge responses [“(comes from) Texas,” “(cycled for) US Postal,” “(had) cancer”) were given beforehand. Participants were then again given the opportunity to recall the correct answer within a time limit of 5 s (henceforth *late recall*). The start and stop of each segment of the recall trial were indicated over the speakers and a tone marked the presentation of a new trial. Order of questions was the same for all participants.

During multiple-choice recognition, questions occurred in the same order as during the recall task. Four alternatives per question were presented in the same order for each participant, and each alternative (labeled A–D) was correct equally often. The time limit for 64 multiple-choice trials was 10 min.

## Results

One potentially ambiguous question was removed from the data, leaving 63 questions in total. One participant who had misunderstood instructions was removed, leaving 25 participants.

### Recall and Recognition Memory

Mean initial recall accuracy was 16.1% (*SD* = 0.1) rising marginally to 18.7% (*SD* = 0.1) at late recall. Trials with correct recall during either recall attempt were removed from subsequent analyses.

Mean recognition for unrecalled answers was 33.7% correct (*SD* = 0.1), which is significantly above the chance guessing level of 25% [*t*(24) = 5.90, *p* (two-tailed) < 0.0001, *r* = 0.77].

### Accuracy of Partial Knowledge

Partial knowledge was scored by two independent raters. Because participants could report up to six items of information per question, maximum score per trial was 6 (1 point per correct item).

Items were scored as correct if they contained fragments of the correct target (e.g., correctly reporting its first letter) or if they referred to other varieties of partial knowledge. For example, for the question “Which actor plays the main character in the TV series Frasier?”, the correct labeling of an attribute of this actor (e.g., “balding”), or comments such as “also plays in the TV series Cheers,” would both gain 1 point.

The mean number of partial knowledge items reported per question was 1.3 (*SD* = 0.6). The two raters agreed on the total score for *correct* partial knowledge items on 73.5% of trials. For remaining trials, they discussed discrepant items and agreed on a score. Mean number of *correct* partial knowledge items was 0.5 per question (*SD* = 0.2).

Note that the number of correct or incorrect items reported on any given trial had a different distribution than in the one study by Koriat (1993, Experiment 1) to previously report this information. In our experiment, only a minority of trials (21%) were associated with 2 or more items of correct partial knowledge, as was the case for incorrect partial knowledge (20%). By comparison, 72% of trials in Koriat’s experiment contained two or more correct partial knowledge items, and only 9% contained more than two items of incorrect partial knowledge. In our study, no partial knowledge at all was reported on 35% of all trials (see **Table 1**)

**Table 1:** Total number of trials (pooled across participants) on which reported partial knowledge contained various possible combinations of incorrect and correct knowledge.

Number of incorrect items	Number of correct items					
	0	1	2	3	4	5	6
0	449	122	48	19	10	0	0
1	213	114	37	11	0	1	0
2	117	38	13	4	0	0	0
3	49	15	0	1	0	0	0
4	12	2	1	0	0	0	0
5	2	0	0	0	0	0	0
6	0	0	0	0	0	0	0

### Partial Knowledge and FoK

**FIGURE 1:**
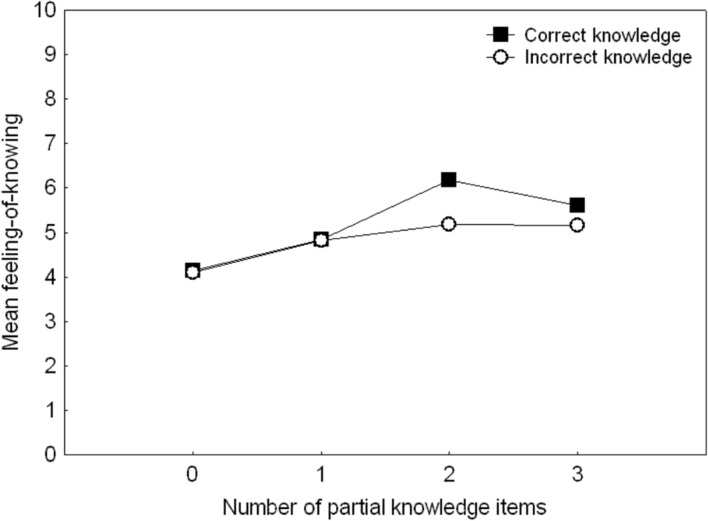
**The relation between FoK rating and number of reported partial knowledge items, plotted separately for correct and incorrect partial knowledge.** Data for more than three items is not plotted due to insufficient number of observations.

**Figure 1** illustrates mean FoK rating for each level of partial knowledge, with separate plots for correct versus incorrect partial knowledge. Note that the two curves are calculated from the same trials, since for a given FoK score, any combination of correct and/or incorrect partial knowledge may have been reported. FoK appears to increase as either correct or incorrect partial knowledge rise from zero to one, but there is an indication that the increment in FoK with further increase in partial knowledge might be different for correct versus incorrect knowledge.

For each participant Pearson’s *r* was calculated for the correlation between FoK rating (1–10) on each trial and the number of *correct* partial knowledge items (0–6) reported on that trial. A separate correlation was similarly calculated for *incorrect* partial knowledge. For correct partial knowledge the mean correlation of 0.22 (*SD* = 0.14) was significantly higher than 0 [*t*(24) = 7.50, *p* (two-tailed) < 0.0001, *r* = 0.84], as was the mean correlation of 0.14 (*SD* = 0.14) for incorrect knowledge [*t*(24) = 5.03, *p* (two-tailed) < 0.001, *r* = 0.72].

Because **Figure 1** indicates a comparable increase in FoK from zero to one item of partial knowledge, but that increases beyond one such item might have different influences on the development of FoK for correct versus incorrect items, modified correlation analyses were then conducted. For correct partial knowledge, trials without any *correct* partial knowledge were excluded. Similarly, the correlation for incorrect partial knowledge was conducted after excluding trials without any *incorrect* knowledge. For correct partial knowledge, the mean positive correlation with FoK (*M* = 0.18, *SD* = 0.26) remained significant [*t*(23) = 3.53, *p* (two-tailed) < 0.01, *r* = 0.59]. However, for incorrect knowledge, the mean correlation (*M* = 0.01, *SD* = 0.24) no longer differed from 0 [*t*(22) = 0.20, *p* (two-tailed) = 0.84, *r* = 0.04].

In other words, the initial correlation between the amount of incorrect partial knowledge and FoK appears to be due to the influence of reporting one item rather than none. In contrast to correct partial knowledge, reporting more than one item of incorrect partial knowledge does not increase FoK any further. Shapiro–Wilk tests showed this null result for incorrect knowledge cannot be attributed to violated normality assumptions for parametric. Since there were more trials with incorrect than correct items, right across the range of number of reported items (see **Table 1**), the null result for incorrect items is also unlikely to be a Type II error.

### Partial Knowledge and Recognition

The correctness of partial knowledge showed a robust effect on recognition accuracy. The number of correct partial knowledge items was significantly greater on correct (*M* = 0.61, *SD* = 0.28) than incorrect (*M* = 0.45, *SD* = 0.19) recognition trials [*t*(24) = 3.59, *p* (two-tailed) < 0.01, *r* = 0.59]. Conversely the number of incorrect partial knowledge items was greater on incorrect (*M* = 0.55, *SD* = 0.32) than correct (*M* = 0.41, *SD* = 0.35) recognition trials [*t*(24) = 2.39, *p* (two-tailed) < 0.05, *r* = 0.44].

### FoK and Recognition

The relationship between FoK and recognition accuracy was expressed for each participant as the signal detection theory (SDT) statistic A_z_ to minimize the influence of response biases to give high or low FoK ratings. A_z_ is calculated from recognition performance (correct versus incorrect) across the range of different FoK ratings, and corresponds to the area under the receiver operating characteristic (ROC) curve (Macmillan and Creelman, 1991; Norman and Price, 2015). An A_z_ score of 1 indicates a perfect positive relationship between FoK and recognition performance, and a score of 0.5 indicates random responding. The mean A_z_ score was 0.55 (*SD* = 0.09), which is significantly above the 0.5 chance level [*t*(24) = 2.62, *p* (two-tailed) = 0.02, *r* = 0.47]. Overall, FoK was therefore predictive of recognition accuracy.

However, further analysis showed that FoK only predicted recognition performance with above-chance accuracy on trials where there was some correct partial knowledge. For each participant, two additional A_z_ scores were calculated between FoK and recognition performance. One included only incorrect recall trials on which some correct partial knowledge was reported (mean number of trials = 17.4, *SD* = 6.1), and one included only those trials where no correct partial knowledge was reported (mean number of trials = 33.7, *SD* = 7.9). For trials lacking any correct partial knowledge, A_z_ (*M* = 0.51, *SD* = 0.12) was not different from chance [*t*(24) = 0.23, *p* (two-tailed) = 0.82], which indicates non-predictive FoK. In contrast, for trials where correct partial knowledge was reported (*M* = 0.59, *SD* = 0.12), FoK was a significant predictor of recognition [*t*(21) = 3.53, *p* (two-tailed) < 0.01, *r* = 0.61]. A_z_ scores for these two types of trial also differed from each other on a related *t*-test [*t*(21) = 2.66, *p* (two-tailed) < 0.05, *r* = 0.50)].

## Discussion

Using naturalistic general knowledge questions as stimuli, this study replicates an established finding that when people cannot recall a memory item, FoK ratings moderately predict subsequent recognition accuracy. The novelty of our study was to make two modifications to the procedure typically used in this type of experiment to test the relation of FoK ratings to partial knowledge: on each trial, we measured FoK before partial knowledge was reported, and free reports of partial knowledge were less constrained than in most previous studies.

### The Relation between FoK and Partial Knowledge

Our own study broadly replicated an apparent influence of partial knowledge on FoK in a study that used general knowledge questions as stimuli. Importantly, we also showed that this holds even though partial knowledge was measured *after* FoK. This demonstrates that any influence of partial knowledge on FoK is not restricted to situations where people could simply base their FoK rating on the amount of partial knowledge that they have just reported, as in previous studies which asked for the information to be reported first (Koriat, 1993, 1995). It is also therefore more supportive of Koriat’s (2000, 2007) suggestion that FoKs are intuitive, *experience-based metacognitive judgments* (see also Norman, 2002; Price, 2002; Price and Norman, 2008; Norman et al., 2010) as opposed to deliberative evaluations of the amount and retrieval speed of partial knowledge.

We found that FoK increased monotonically with the amount of both correct *and* incorrect partial knowledge. Both correct and incorrect partial knowledge were positively related to the strength of FoK as long as analyses included, respectively, trials without any correct or incorrect partial information. When these subsets of trials were removed, correct partial knowledge was still a significant predictor of FoK, but incorrect partial knowledge was not. Therefore it seems that FoK increases with increasing amounts of correct partial knowledge, and that the presence—but not the amount—of incorrect partial knowledge also boosts FoK. This is inconsistent with the findings of Koriat (1993, 1995), and more in line with later studies that looked at the relationship between fragment knowledge, FoK and memory for newly acquired knowledge (Dunlosky et al., 2005; Thomas et al., 2011, 2012).

One interpretation is that as the reported amount of incorrect partial knowledge increases, it is more likely to reflect pure guesses. However, this is unlikely to be the case since incorrect partial knowledge negatively influenced recognition. If incorrect items were thought to be correct, they would act as misleading cues and actively impair selection of the right answer, but if incorrect items were known to be guesses they should have a more neutral impact on subsequent recognition.

The asymmetry we found between the influence of correct and incorrect partial knowledge is at odds with existing formulations of the accessibility hypothesis, and with Koriat’s original findings. However, we suggest that it is actually *more* consistent with Koriat’s central claim that FoK is an experience-based metacognitive judgment that is influenced by activated partial knowledge. When partial knowledge is activated via spreading activation in semantic networks, there will be variation in the representational distance between this knowledge and the target domain activated by the general knowledge question. On average, partial knowledge that is closest to the target domain will be most highly activated, and is also most likely to be correct—i.e., rated as closely relevant by the experimenters. Items of partial knowledge that are incorrect will therefore on average contribute less activation to whatever pooled global signal underlies the FoK. And increases in the number of incorrect items will have less influence than correct knowledge on the strength of the FoK. By contrast, the fact that incorrect and correct partial knowledge were previously found to be equally influential on FoK ratings supports our suspicion that, in other studies, these ratings were heavily based on what Koriat (2000, 2007) has referred to as more deliberative *information-based metacognitive judgments* of reported partial knowledge.

Why would activation of incorrect knowledge nevertheless appear to make at least some contribution to FoK in a manner that is independent of the amount of knowledge reported? Possibly this reflects the contribution that general familiarity with the topic of the question has on FoK (the cue familiarity hypothesis, Reder and Ritter, 1992; Metcalfe et al., 1993). This contribution is well established and is integrated into broader theoretical models of FoK (e.g., Koriat and Levy-Sadot, 2001; Liu et al., 2007). Cue familiarity could contribute to FoK even when only incorrect partial knowledge was activated, accounting for the apparent influence of one or more incorrect items of knowledge. However, greater degrees of cue familiarity need not necessarily be associated with increments in the reported amount of incorrect partial knowledge.

### The Relation between Recognition and Partial Knowledge

The accuracy of partial knowledge was significantly related to subsequent recognition performance, and FoK only predicted recognition with above-chance accuracy on trials where some correct partial knowledge had been reported. Although this has not consistently been demonstrated in the past (Koriat, 1993, 1995), it is in line with theories that stress the role of partial knowledge in FoK, and also with empirical findings from experimental situations in which semantically meaningful information was accessible (Thomas et al., 2011, 2012). The reason why we found such a relationship may also have to do with our measurement procedure. In our study, partial knowledge was broadly measured using open-ended reports of all target-related knowledge. This argues that general semantic ingredients contribute to FoK and subsequent recognition. In contrast, in the study where Koriat (1995, Experiment 3) used general knowledge questions as stimuli, he measured very specific types of partial knowledge such as single letters and number of syllables. The demand to generate such specific fixed category responses might increase the discrepancy between the partial knowledge reported and the partial knowledge responsible for genuinely predictive FoK judgments and successful recognition performance. Our results can therefore be regarded as compatible with those of Thomas et al. (2011, 2012).

Another effect of the rather narrow response categories in Koriat’s original studies is that they are more likely to encourage guessing, since they decrease the likelihood that genuine partial knowledge can be reported, and provide an easy set of available response alternatives to generate guesses. In Koriat’s (1995, Experiment 3) study using general knowledge questions, guessing was even explicitly encouraged. If a large proportion of incorrect partial knowledge items derive from guesses, then incorrect items in general can be considered largely as random noise. As pointed out earlier, the difference in recognition performance that follows reports of incorrect partial knowledge items versus predictive correct items will therefore be less than when incorrect items are genuinely misleading cues that actively depress recognition performance. Additionally, the predictive accuracy of correct partial knowledge may be reduced since the intentional generation of guessed responses could interfere with retrieval of correct partial knowledge during the recall phase of an experiment.

Note that these problems were minimized in the one experiment where Koriat (1993, Experiment 1) found the predicted relationship between recognition accuracy and correct/incorrect partial knowledge. First, that experiment used meaningless letter strings where the range of potential partial knowledge is smaller than for general knowledge questions and is therefore more likely to be captured by narrow response categories. Second, the experiment discouraged guessing by respectively rewarding or punishing reports of correct or incorrect partial knowledge.

Our procedure was designed to reduce the likelihood that FoK ratings reflected explicit evaluations of the amount of partial knowledge that was deliberately retrieved and reported. Still, one cannot completely rule out the possibility that the instruction to report partial knowledge on a trial-by-trial basis may, at a general level, influence people’s tendency to retrieve partial information, even before being explicitly instructed to do so. One possible consequence of such evaluation could be an increase in the frequency of undetected high-confidence errors. However, we find it unlikely that a 10-s recall phase would have been sufficient to both attempt retrieving the target information as well as explicitly retrieving and evaluating partial knowledge. Moreover, the aim of measuring FoKs before rather than after partial knowledge was also not to eliminate any potential influence of explicitly generated partial knowledge, but instead to rule out the possibility that the influence of partial knowledge on FoK is limited to situations in which people have been specifically instructed to report certain forms of partial knowledge before rating FoK.

## Conclusion

According to Koriat’s (1993, 1995) *accessibility hypothesis*, FoK increases with the amount of partial knowledge that can be reported during the initial recall attempt, regardless of the accuracy of this knowledge. Even though the influence of partial knowledge on FoK is also confirmed by later studies using word or picture stimuli (Dunlosky et al., 2005; Thomas et al., 2011, 2012), existing studies disagree as to whether the influence of partial knowledge on FoK depends on its accuracy. By applying a procedure that avoided some methodological limitations of Koriat’s original studies, we found support for the hypothesis that FoK is partly based on the kind of intuitive assessment of target-related partial knowledge that has been referred to as experience-based metacognitive judgment (Koriat, 2007). Both of our procedural modifications appeared to be relevant: i.e., the use of a very open-ended measure of partial knowledge, and measuring FoK before partial knowledge. Together they reduce the possibility that FoK ratings were based on a deliberative summing up and evaluation of partial knowledge from artificially constrained categories that might encourage participants to generate spurious partial knowledge reports.

## Author Contributions

EN had the main responsibility for data analysis and the write-up. OB, ØJ, and SKM participated in the design and data analysis, and collected the data. They also contributed to the data analysis and interpretation of results and provided input on the draft. MCP contributed to the planning of the study, the interpretation of results, and also in the write-up of the paper.

## Conflict of Interest Statement

The authors declare that the research was conducted in the absence of any commercial or financial relationships that could be construed as a potential conflict of interest.
